# Comparative secretome of white‐rot fungi reveals co‐regulated carbohydrate‐active enzymes associated with selective ligninolysis of ramie stalks

**DOI:** 10.1111/1751-7915.13647

**Published:** 2020-08-14

**Authors:** Chunliang Xie, Wenbing Gong, Zuohua Zhu, Yingjun Zhou, Chao Xu, Li Yan, Zhenxiu Hu, Lianzhong Ai, Yuande Peng

**Affiliations:** ^1^ Institute of Bast Fiber Crops Chinese Academy of Agricultural Sciences Changsha 410205 China; ^2^ Shanghai Engineering Research Center of Food Microbiology School of Medical Instrument and Food Engineering University of Shanghai for Science and Technology Shanghai 200093 China

## Abstract

In the present research, *Phanerochaete chrysosporium* and *Irpex Lacteus* simultaneously degraded lignin and cellulose in ramie stalks, whereas *Pleurotus ostreatus* and *Pleurotus eryngii* could depolymerize lignin but little cellulose. Comparative proteomic analysis of these four white‐rot fungi was used to investigate the molecular mechanism of this selective ligninolysis. 292 proteins, including CAZymes, sugar transporters, cytochrome P450, proteases, phosphatases and proteins with other function, were successfully identified. A total of 58 CAZyme proteins were differentially expressed, and at the same time, oxidoreductases participated in lignin degradation were expressed at higher levels in *P. eryngii* and *P. ostreatus*. Enzyme activity results indicated that cellulase activities were higher in *P. chrysosporium* and *I. lacteus*, while the activities of lignin‐degrading enzymes were higher in *P. eryngii* and *P. ostreatus*. In addition to the lignocellulosic degrading enzymes, several proteins including sugar transporters, cytochrome P450 monooxygenases, peptidases, proteinases, phosphatases and kinases were also found to be differentially expressed among these four species of white‐rot fungi. In summary, the protein expression patterns of *P. eryngii* and *P. ostreatus* exhibit co‐upregulated oxidoreductase potential and co‐downregulated cellulolytic capability relative to those of *P. chrysosporium* and *I. lacteus*, providing a mechanism consistent with selective ligninolysis by *P. eryngii* and *P. ostreatus*.

## Graphical abstract

*P. eryngii* and *P. ostreatus* protein expression patterns exhibit increased oxidoreductase potential and diminished cellulolytic capability. Co‐regulated carbohydrate‐active enzymes were associated with selective ligninolysis of ramie stalks.

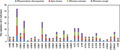

## Introduction

Eight million tons of agricultural by‐products rich in lignocellulose are produced in China every year (Venanzi *et al*., 2018). These by‐products can result in environmental pollution and a serious waste of resources for their disposal (Fasolato *et al*., 2016). At present, methods for comprehensive utilization of these lignocellulosic stalks have been developed, which have greatly improved the degree of utilization of stalks (Kim, 2018). One example of such improved utilization is the use of stalks as feed stocks for producing ethanol (Jiang *et al*., 2017). However, the lignin content of native lignocellulosic biomass is highly resistant to enzymatic hydrolysis, resulting in low cellulose conversion. Therefore, lignin must be selectively degraded in an effective pretreatment process to improve enzymatic saccharification (Guo *et al*., 2018). To date, there has been extensive research into the best pretreatment methods, including physical, chemical or biological degradation of lignin (Kim, 2018). Biological pretreatment has garnered considerable attention in recent years because it is a mild, safe and environmental‐friendly way to remove lignin from lignocellulosic materials (Paudel *et al*., 2017).

Although white‐rot fungi are the most promising microorganisms used for lignin degradation, they differ in their gross morphological patterns of decay (da Silva *et al*., 2017). *Phanerochaete chrysosporium* simultaneously degrades cellulose, hemicellulose and lignin, whereas other typical white‐rot fungi, such as *Pleurotus ostreatus*, *Coriolus versicolor* and *Ceriporiopsis subvermispora*, degrade lignin before degrading cellulose (Fernandez‐Fueyo *et al*., 2012). Pretreatment of wheat straw with *C. subvermispora* showed that after 35 days, the degradation ratio of lignin in wheat straw was 55% and the conversion efficiency of cellulose was 66.61% (Cianchetta *et al*., 2014). In addition, after pretreatment of rice straw by *P. ostreatus* for 60 days, the lignin degradation ratio was 52%, while 83% of cellulose was retained, indicating that *P. ostreatus* also had a selective ability to degrade lignin (Taniguchi *et al*., 2005). To investigate the mechanistic basis for such selective ligninolysis, Fernandez‐Fueyo *et al*. conducted a comparative transcriptomic analysis of *C. subvermispora* and *P. chrysosporium* (Fernandez‐Fueyo *et al*., 2012). Several genes, including those encoding manganese peroxidase (MnP) and laccases, were found to be differentially expressed between the *C.subvermispora* and *P. chrysosporium* transcriptomes. However, that study only compared transcriptomic differences between two species of white‐rot fungi. With the completion of the genome sequences of a variety of white‐rot fungi and advances in quantitative proteomics technologies, analyses of the expression, mechanisms and regulation of enzymes involved in the lignin degradation pathways from a systems biology point of view will be emphasized in the near future (Kameshwar and Qin, 2016).

Ramie (*Boehmeria nivea* L. Gaud) is an important natural fiber crop that is widely planted in China, India and other Southeast Asian and Pacific Rim countries (Luan *et al*., 2018). Large quantities of ramie fibres are extracted from ramie stalks every year (Meng *et al*., 2018). The massive ramie stalks are a promising renewable source of lignocellulose and can be used as animal feed, fertilizer, fuel ethanol and a substrate for mushroom cultivation (Xie *et al*., 2017). In our previous research, we used ramie stalks to cultivate *Pleurotus eryngii* and reached a biological conversion efficiency of 71% (Xie *et al*., 2016a). In addition, we obtained a global profile of the secretome of *P. eryngii* cultivated in ramie stalk medium. Diverse enzymes, including cellulases, hemicellulases, pectinase, ligninase, proteases, peptidases and phosphatases, implicated in lignocellulose degradation were detected (Xie *et al*., 2016a).

The lignin content of ramie stalks is approximately 18.1% by weight and directly affects enzymatic saccharification during the process of ethanol production (Xie *et al*., 2016b). Therefore, it has become necessary to screen for white‐rot fungi that can carry out selective ligninolysis to use for biological pretreatment. Thus, we screened for fungal species that could selectively degrade lignin from ramie stalks. Further, to better understand the molecular mechanism of this selective ligninolysis of ramie stalks at the level of protein expression, iTRAQ labelling combined with liquid chromatography/tandem mass spectrometry (LC‐MS/MS) was used to investigate the differential expression of lignin degradation‐related proteins between *P. eryngii* and *P. ostreatus*, which selectively degrade lignin, and *P. chrysosporium* and *I. lacteus*, which non‐selectively degrade lignin. A proposed molecular mechanism of selective degradation of lignin by *P. eryngii* (CICC50126) and *P. ostreatus* (bio‐67015) was also discussed.

## Results and discussion

### Lignocellulose degradation of ramie stalks by *P. chrysosporium, I. lacteus, P. eryngii* or *P. ostreatus*

Lignocellulose, including cellulose, hemicellulose and lignin, is the most abundant renewable biomass in the world. Bioconversion of lignocellulosic biomass plays important roles in exploiting value added products (Zhu *et al*., 2017). Lignin makes lignocellulose recalcitrant to degradation because of its highly irregular and heterogeneous biopolymer (Schutyser *et al*., 2018). Currently, white‐rot fungi are considered as the most important lignin‐degrading organisms (Knop *et al*., 2015). Degradation of lignin by white‐rot fungi can occur in two ways: (i) fungi secrete enzymes that selectively remove lignin polymers and leave cellulose intact; or (ii) fungi degrade lignin and cellulose simultaneously (van Kuijk *et al*., 2015).

**Fig. 1:**
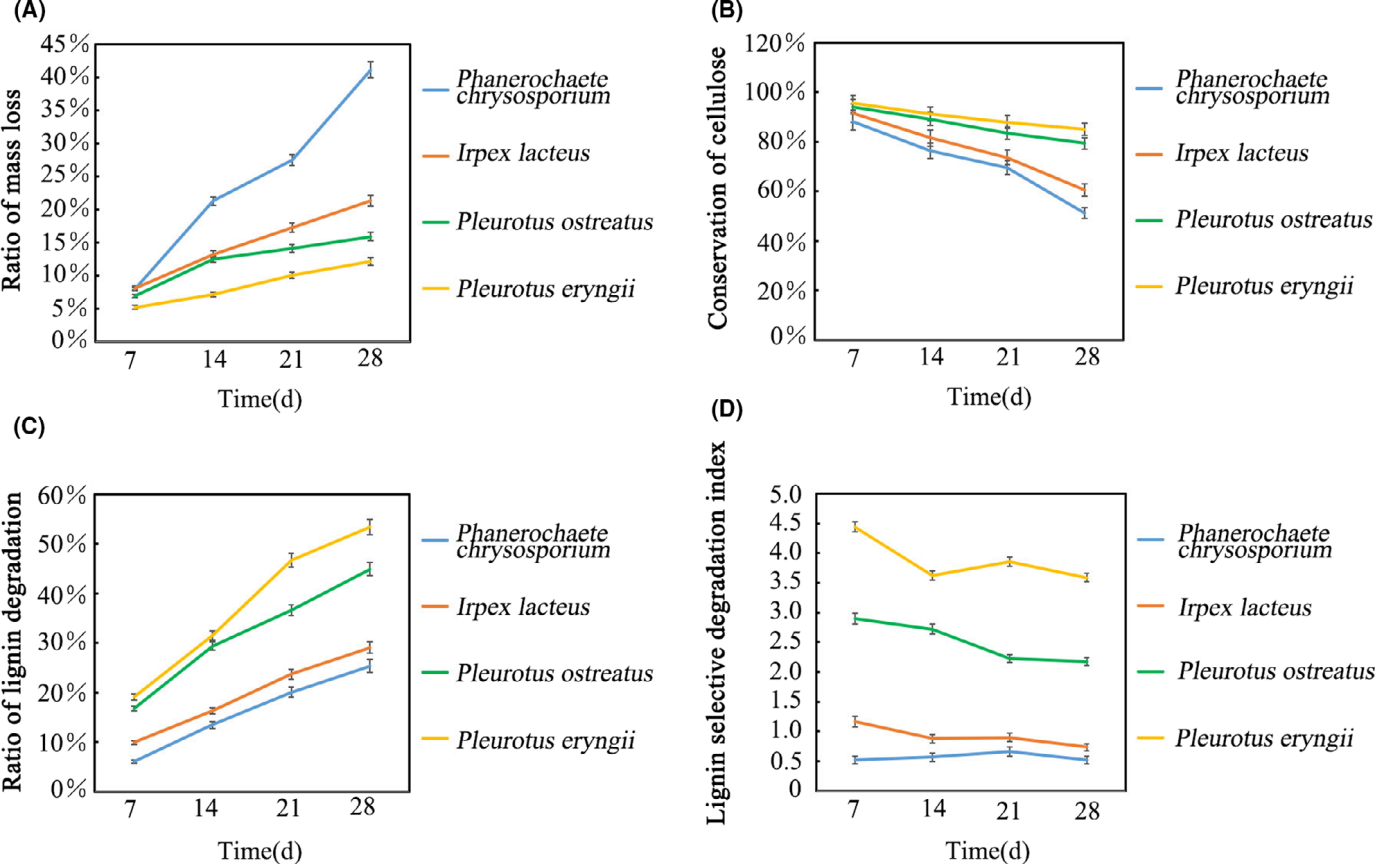
The ratios of weight loss, conservation of cellulose, lignin degradation and lignin‐selective degradation index after treatment of *P. chrysosporium*, *I. lacteus*, *P. eryngii* and *P. ostreatus* in 7, 14, 21 and 28 days. From all treatments at 21 or 28 days, the weight loss and lignin‐selective degradation index of *P. chrysosporium* had significant difference with *P. eryngii* and *P. ostreatus*, while conservation of cellulose and lignin degradation ratios of *P. eryngii* and *P. ostreatus* were significantly higher than *I. lacteus* and *P. chrysosporium* (*P* < 0.01).

Degradation of lignin is the limiting step for enzymatic hydrolysis of cellulose in ramie stalks. The lignin‐selective degradation index can be measured by determining lignin degradation with simultaneous conservation of cellulose. In the present research, ramie stalks were treated with *P. chrysosporium*, *I. lacteus*, *P. eryngii* and *P. ostreatus* for 7, 14, 21 or 28 days. Figure 1 showed that degradation of cellulose in ramie stalks treated with *P. eryngii* and *P. ostreatus* is lower than that with *I. lacteus* or *P. chrysosporium*, while the lignin degradation caused by *P. eryngii* is greater than that caused by other fungi. The lignin‐selective degradation indices for *P. chrysosporium*, *I. lacteus*, *P. eryngii* and *P. ostreatus* at 21 days were 0.66, 0.89, 3.86 and 2.22, respectively. These results indicated that *P. eryngii* and *P. ostreatus* had more selective ability to degrade lignin in ramie stalks, while *P. chrysosporium* and *I. lacteus* degrade large amounts of cellulose, hemicellulose and lignin due to their non‐selective pattern of ligninolysis. Previous study reported similar degradative capability of *P. chrysosporium* and *P. ostreatus. P. chrysosporium* was found to cause higher amount of lignin and carbohydrate fraction loss. *P. ostreatus* have shown lower cellulose and hemicellulose loss, while lignin removal reached 40% (Taniguchi *et al*., 2005; Shrivastava *et al*., 2011).

### Enzymatic hydrolysis of ramie stalks treated with *P. chrysosporium, I. lacteus, P. eryngii* or *P. ostreatus*

**Fig. 2:**
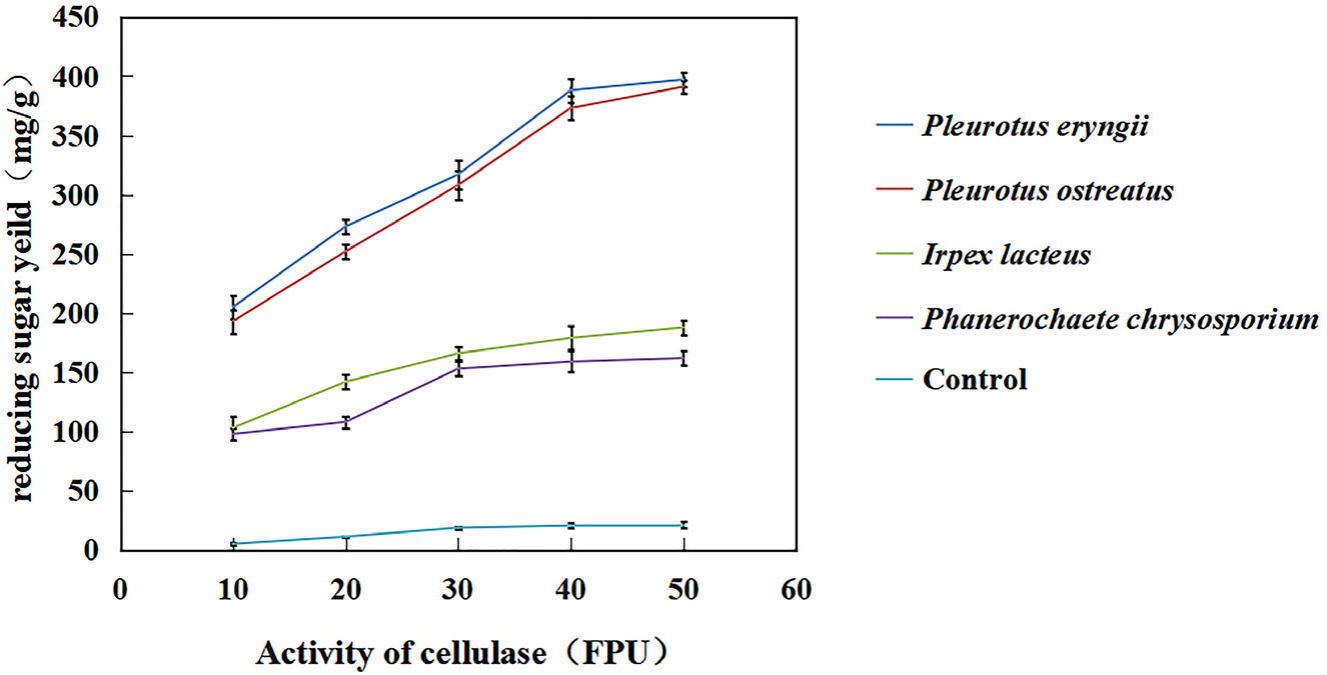
The reducing sugar yield of four white‐rot fungi treated ramie stalks after 48‐h enzymatic hydrolysis with cellulase. The yield of reducing sugars of *P. eryngii* has no statistically significant difference with *P. ostreatus*, while was significantly higher than *I. lacteus* and *P. chrysosporium* (*P* < 0.01).

Lignin is a complex biopolymer which is considered as a main barrier against enzymatic degradation. The unproductive binding of cellulase and lignin is considered to be one of the important reasons for reducing the efficiency of enzymatic hydrolysis (Alvarez et al., 2016). To evaluate the effect of selective ligninolysis on the subsequent enzymatic hydrolysis of cellulose, the reducing sugar yield of ramie stalks pretreated with *P. chrysosporium, I. lacteus, P. eryngii* or *P. ostreatus* was determined after 48‐h hydrolysis with cellulase, using untreated ramie stalks as control. Figure 2 showed that the yield of reducing sugars of *P. eryngii* was 388 mg g^−1^ after addition of 40 FPU g^−1^ cellulase when treated for 21 days, which was significantly higher than that of *I. lacteus* (179 mg g^−1^) or *P. chrysosporium* (159 mg g^−1^) but was not statistically significant different from that of *P. ostreatus* (373 mg g^−1^). As expected, the control ramie stalks that were not pretreated had a very low hydrolysis ratio (20.5 mg g^−1^). Previous studies reported the release of 330 mg g^−1^ of sugar from rice straw fermented with *P. ostreatus* and release of 402 mg g^−1^ sugar from *Prosopis Juliflora* fermented with *Pycnoporus cinnabarinus*. These results indicated that pretreatment of substrates with white‐rot fungi can improve enzymatic saccharification compared with those untreated substrates (Consolacion *et al*., 2016). Our study showed that *P. ostreatus* and *P. eryngii* were suitable for pretreatment of ramie stalks prior to hydrolysis by cellulases due to its selective ligninolysis ability. This may be attributed to the increase of the effective surface area, the decrease of polymerization degree, the separation of lignin carbohydrate bond and the modification of lignin, so as to facilitate the accessibility of enzymatic hydrolysis substrate (Juan and Estrella, 2019).

### Enzyme activities of *P. chrysosporium, I. lacteus, P. eryngii* and *P. ostreatus* during lignin degradation in ramie stalks

**Fig. 3:**
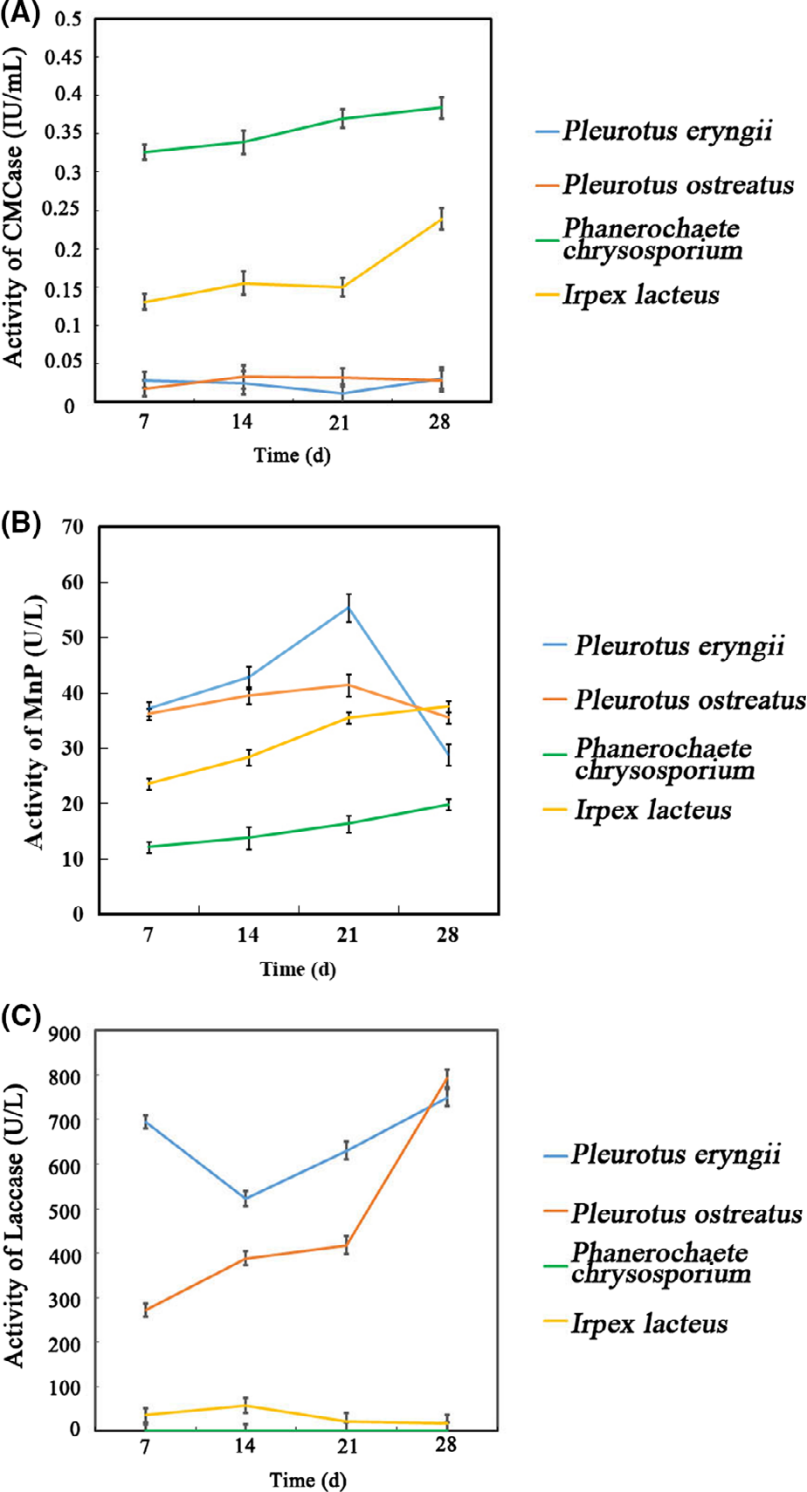
CMCase, MnP and laccase activities of *P. chrysosporium* and *I. lacteus* had significant difference with *P. eryngii* and *P. ostreatus* during ramie stalks lignin degradation process at 7, 14 or 21 days (*P* < 0.01).

The difference in selective ligninolysis by *P. chrysosporium*, *I. lacteus*, *P. eryngii* and *P. ostreatus* could be attributed to the different enzymatic activities observed during growth on ramie stalks. In this study, carboxymethyl cellulose enzyme (CMCase), MnP and laccase activities were detected during lignin degradation of ramie stalks by *P. chrysosporium, I. lacteus, P. eryngii and P. ostreatus*. Figure 3 showed that the CMCase activity of *P. chrysosporium* was significantly higher than that of the other three fungi. Peak CMCase activities of *P. chrysosporium* and *I. lacteus* were 0.384 and 0.239 IU ml^−1^, respectively, at 28 days, while the CMCase activities of *P.ostreatus* and *P. eryngii* were lower than 0.1 IU ml^−1^ at that time point. Not surprisingly, ramie stalks pretreated with *P. chrysosporium* exhibited considerable cellulose degradation. The peak MnP activity value of *P. chrysosporium* (19.76 U l^−1^) was appeared in at 28 days, while the peak MnP activities of *P. eryngii* (55.32 U l^−1^) and *P. ostreatus* (41.37 U l^−1^) were detected at 21 days. Figure 3 showed that laccase activity of *P. ostreatus* and *P. eryngii* also peaked at 28 days, while *P. chrysosporium* exhibited no laccase activity. Interestingly, laccase activity of *P. eryngii* remained high during the entire incubation period (694 U l^−1^ at 7 days, 522 U l^−1^ at 14 days, 630 U l^−1^ at 21 days and 749 U l^−1^ at 28 days) as compared with the other three fungal species tested here. Thus, the lower cellulase activities and higher laccase activities resulted in greater selective degradation of lignin and retention of cellulose upon treatment of ramie stalks with *P. ostreatus* or *P. eryngii*.

### Global analysis of the *P. chrysosporium, I. lacteus, P. eryngii* and *P. ostreatus* secretomes

**Fig. 4:**
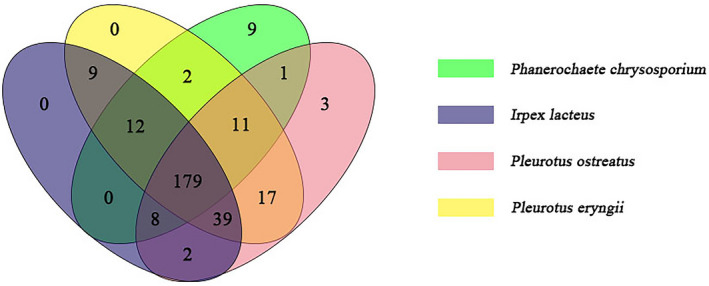
The total extracellular protein identified in the *P. chrysosporium*, *I. lacteus*, *P. eryngii* and *P. ostreatus* secretome.

The difference in the degradation pattern of substrate by these four white‐rot fungi could be attributed to the different enzyme profile observed during growth on ramie stalks. In order to understand the molecular mechanism of selective ligninolysis of ramie stalks, the secretomes of *P. chrysosporium, I. lacteus, P. eryngii and P. ostreatus* were analysed by iTRAQ labelling combined with LC‐MS/MS. As showed in Fig. 1D, the selective degradation index of lignin tends to be stable at 21 days for all of the four white‐rot fungi. So the diversity and relative abundance of the secreted proteins at 21 days of growth on ramie stalk medium were compared. A total of 222, 249, 269 and 260 extracellular proteins were identified in the *P. chrysosporium*, *I. lacteus*, *P. eryngii* and *P. ostreatus* secretomes, respectively (Tables S1–S5). As shown in Fig. 4, 43, 70, 90 and 81 proteins were only detected in the secretomes of *P. chrysosporium*, *I. lacteus*, *P. eryngii* and *P. ostreatus*, respectively. Interestingly, 179 proteins were expressed by all four of these fungal species, revealing a similar effect of ramie stalks on the secretion of proteins by each species. In addition, 20% of the expressed proteins were varied in the secretomes of these four fungi. This reveals that the types of extracellular enzymes produced by white‐rot fungi were mainly influenced by the culture medium and also were related to the unique strains.

The proteins identified in the secretomes of *P. chrysosporium, I. lacteus, P. eryngii and P. ostreatus* grown on ramie stalk medium were functionally classified as carbohydrate‐active enzymes (CAZymes), sugar transporters, intracellular metabolism proteins, cytochrome P450s, proteases, phosphatases and proteins with other functions. The overall proteomic profiles of the *P. eryngii* and *P. ostreatus* secretomes were similar (Tables S4 and S5). The highest diversity of CAZyme proteins was found in the *P. eryngii* and *P. ostreatus* secretomes, while sugar transporter, proteases and phosphatases were similarly abundant among these four fungi (Tables S1–S5).

### Differentially expressed CAZyme proteins in the *P. chrysosporium, I. lacteus, P. eryngii* and *P. ostreatus* secretomes

**Fig. 5:**
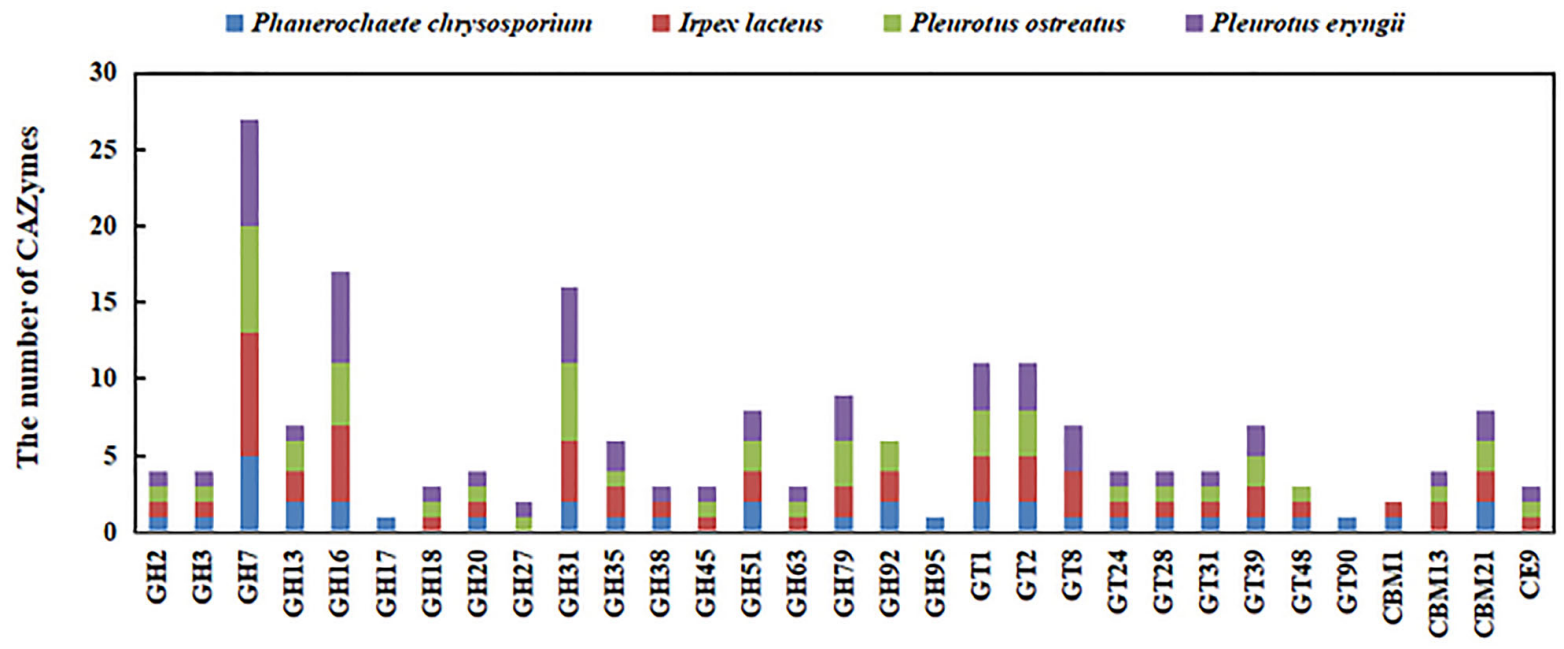
CAZymes identified in *P. chrysosporium*, *I. lacteus*, *P. eryngii* and *P. ostreatus* secretome.

An iTRAQ‐labelling quantitative proteomics approach was used to analyse the differentially expressed proteins in the secretomes of *P. chrysosporium, I. lacteus, P. eryngii* and *P. ostreatus*. Our results showed special emphasis on CAZymes in the secretomes of these white‐rot fungi. In this study, by querying differentially expressed proteins against the Carbohydrate‐Active Enzyme database (CAZy), 58 proteins were identified as CAZymes. Among these CAZymes, 19 glycoside hydrolases (GHs), 10 glucosyl transferases (GTs), 25 auxiliary activities (AAs) and 4 carbohydrate‐binding modules (CBMs) were identified, while no carbohydrate esterases (CEs) or polysaccharide lyases (PLs) were found (Table 1). Closer inspection of particular CAZyme families revealed clear differences in the number and distribution of CAZymes among these four white‐rot fungal species (Fig. 5). For instance, GH17, GH95, CBM1 and GT90 were absent from the *P. eryngii* and *P. ostreatus* secretomes, while laccases were absent from the *P. chrysosporium* secretome. To highlight comparisons of the cellulolytic system, compared with the *P. chrysosporium* and *I. lacteus* secretomes, Table 1 showed that GH2, GH3, GH7, GH20, GH31, GH63, CBM21, GT1, GT2, GT24, GT39, cellobiose dehydrogenase and beta‐galactosidase were less strongly expressed in *P. eryngii* and *P. ostreatus* secretomes. Based on these results, it was suggested that *P. chrysosporium* and *I. lacteus* degrade cellulose, hemicellulose and pectins using a large machinery of GHs. Due to the specific hydrolytic action of these enzymes, large polysaccharide fragments of ramie stalks were mostly released and lost when treated by *P. chrysosporium* and *I. lacteus*.

**Table 1:** Differentially expressed CAZymes proteins in *P. chrysosporium*, *I. lacteus*, *P. eryngii* and *P. ostreatus* secretome.

Accession	Description	*P. chrysosporium*	*I. lacteus*	*P. eryngii*	*P. ostreatus*
R7S0V0	Beta‐galactosidase	2.80 ± 0.14^b^	2.69 ± 0.11^b^	0.55 ± 0.01^a^	0.58 ± 0.03^a^
A0A060STH0	Carbohydrate‐Binding Module Family 1	1.59 ± 0.06^b^	1.76 ± 0.07^b^	0 ± 0^a^	0 ± 0^a^
A0A060SEQ0	Carbohydrate‐Binding Module Family 1	1.96 ± 0.07^b^	1.89 ± 0.08^b^	0 ± 0^a^	0 ± 0^a^
K5WA25	Carbohydrate‐binding module family 21 protein	1.98 ± 0.04^b^	2.21 ± 0.14^b^	0.87 ± 0.02^a^	0.65 ± 0.03^a^
A0A060SC07	Carbohydrate‐Binding Module Family 21 protein	1.86 ± 0.09^b^	2.52 ± 0.11^c^	0.98 ± 0.04^a^	0.97 ± 0.06^a^
Q12661	Cellobiose dehydrogenase	2.99 ± 0.08^c^	2.05 ± 0.05^b^	0.35 ± 0.01^a^	0.65 ± 0.10^a^
Q01738	Cellobiose dehydrogenase	2.54 ± 0.06^b^	2.18 ± 0.12^b^	0.65 ± 0.05^a^	0.39 ± 0.05^a^
Q01599	Glucanase (GH7)	1.87 ± 0.07^b^	2.17 ± 0.10^b^	0.32 ± 0.02^a^	0.27 ± 0.01^a^
Q75NB5	Glucanase (GH7)	2.28 ± 0.11^b^	2.54 ± 0.11^b^	0.24 ± 0.01^a^	0.62 ± 0.15^a^
Q7LHI2	Glucanase (GH7)	2.89 ± 0.08^b^	2.32 ± 0.06^b^	0.66 ± 0.10^a^	0.35 ± 0.11^a^
Q01762	Glucanase(GH7)	2.41 ± 0.12^b^	2.44 ± 0.14^b^	0.36 ± 0.05^a^	0.54 ± 0.02^a^
A5AA53	Glucanase(GH7)	2.62 ± 0.10^b^	2.36 ± 0.12^b^	0.85 ± 0.11^a^	0.62 ± 0.03^a^
A0A060SV04	Glycoside Hydrolase Family 2 protein	1.88 ± 0.05^b^	1.95 ± 0.02^b^	0.28 ± 0.10^a^	0.65 ± 0.03^a^
A0A060SIS9	Glycoside Hydrolase Family 20 protein	2.68 ± 0.06^b^	2.95 ± 0.14^b^	0.55 ± 0.05^a^	0.35 ± 0.02^a^
M2R8M9	Glycoside hydrolase family 3 protein	2.65 ± 0.06^b^	2.87 ± 0.10^b^	0.54 ± 0.05^a^	0.65 ± 0.09^a^
K5UMD6	Glycoside hydrolase family 3 protein	2.44 ± 0.12^b^	2.57 ± 0.10^b^	0.68 ± 0.08^a^	0.72 ± 0.06^a^
M2Q417	Glycoside hydrolase family 31 protein	2.24 ± 0.09^b^	2.52 ± 0.11^b^	0.98 ± 0.10^a^	0.82 ± 0.12^a^
K5WLD7	Glycoside hydrolase family 31 protein	2.68 ± 0.13^b^	2.88 ± 0.13^b^	0.29 ± 0.05^a^	0.35 ± 0.06^a^
K5VQ52	Glycoside hydrolase family 31 protein	2.93 ± 0.14^b^	2.67 ± 0.05^b^	0.79 ± 0.11^a^	0.92 ± 0.08^a^
A0A060SCJ9	Glycoside Hydrolase Family 31 protein	2.18 ± 0.10^b^	2.39 ± 0.07^b^	0.87 ± 0.14^a^	0.81 ± 0.01^a^
A0A060SK35	Glycoside Hydrolase Family 31 protein	2.56 ± 0.12^b^	2.25 ± 0.05^b^	0.77 ± 0.09^a^	0.97 ± 0.13^a^
K5VW03	Glycoside hydrolase family 63 protein	1.98 ± 0.04^b^	2.54 ± 0.14^c^	0.36 ± 0.10^a^	0.58 ± 0.10^a^
K5W2Q0	Glycoside hydrolase family 95 protein	2.24 ± 0.12^b^	1.85 ± 0.05^b^	0 ± 0^a^	0 ± 0^a^
A0A0L6JAQ3	Glycosyl transferase family 1	1.53 ± 0.07^b^	1.89 ± 0.03^b^	1.38 ± 0.04^ab^	0.89 ± 0.09^a^
A0A0L6J6R9	Glycosyl transferase family 1	1.68 ± 0.05^b^	1.59 ± 0.02^b^	0.47 ± 0.01^a^	0.53 ± 0.13^a^
A0A0L6J4U4	Glycosyl transferase family 1	1.75 ± 0.04^b^	1.45 ± 0.03^b^	0.52 ± 0.03^a^	0.44 ± 0.02^a^
M2QJ27	Glycosyltransferase family 2 protein	2.84 ± 0.08^b^	2.45 ± 0.07^b^	0.65 ± 0.08^a^	0.47 ± 0.11^a^
M2QDK4	Glycosyltransferase family 2 protein	2.65 ± 0.08^b^	2.33 ± 0.15^b^	0.85 ± 0.02^a^	0.58 ± 0.06^a^
A0A060SI51	Glycosyltransferase Family 2 protein	2.15 ± 0.06^b^	2.98 ± 0.16^c^	0.67 ± 0.06^a^	0.44 ± 0.03^a^
M2Q5U6	Glycosyltransferase family 24 protein	2.99 ± 0.07^b^	2.45 ± 0.12^b^	0.36 ± 0.01^a^	0.68 ± 0.03^a^
A0A060T0D5	Glycosyltransferase Family 39 protein	2.97 ± 0.09^b^	2.43 ± 0.14^b^	0.74 ± 0.15^a^	0.89 ± 0.04^a^
A0A060S351	Glycosyltransferase Family 39 protein	2.55 ± 0.06^b^	2.68 ± 0.10^b^	0.77 ± 0.01^a^	0.59 ± 0.03^a^
A0A060SSS4	Glycosyltransferase Family 90 protein	1.99 ± 0.03^b^	1.65 ± 0.05^b^	0 ± 0^a^	0 ± 0^a^
R7T1B0	Glyoxal oxidase	0.65 ± 0.03^a^	0.59 ± 0.03^a^	4.54 ± 0.01^c^	3.28 ± 0.19^b^
A0A060SS83	Glyoxal oxidase	0.69 ± 0.06^a^	0.98 ± 0.13^a^	2.17 ± 0.05^b^	1.88 ± 0.18^b^
A0A060SYB0	Glyoxal oxidase	0.77 ± 0.06^a^	0.85 ± 0.06^a^	3.78 ± 0.01^c^	2.54 ± 0.04^b^
R7SNP0	Glyoxylate dehydrogenase	0.68 ± 0.10^a^	0.25 ± 0.03^a^	3.42 ± 0.15^c^	2.21 ± 0.19^b^
R7SMV5	GMC oxidoreductase	0.69 ± 0.08^a^	0.87 ± 0.10^a^	2.84 ± 0.03^c^	1.74 ± 0.23^b^
A0A060SUN5	GMC oxidoreductase	0.58 ± 0.01^a^	0.97 ± 0.05^a^	2.88 ± 0.12^c^	1.72 ± 0.20^b^
A0A060SLW4	GMC oxidoreductase	0.62 ± 0.12^a^	0.75 ± 0.04^a^	3.96 ± 0.13^c^	2.68 ± 0.25^b^
R7RXB0	GMC oxidoreductase	0.39 ± 0.03^a^	0.54 ± 0.03^a^	1.56 ± 0.02^b^	1.69 ± 0.07^b^
A0A0A0RN90	Laccase	0 ± 0^a^	0 ± 0^a^	2.65 ± 0.15^c^	1.71 ± 0.05^b^
O60199	Laccase	0 ± 0^a^	0.25 ± 0.02^b^	2.99 ± 0.11^d^	1.68 ± 0.22^c^
Q96TR4	Laccase	0 ± 0^a^	0.34 ± 0.08^b^	2.65 ± 0.17^c^	2.15 ± 0.03^c^
Q6RYA4	Laccase	0 ± 0^a^	0.26 ± 0.01^b^	2.97 ± 0.18^c^	2.24 ± 0.23^c^
Q6R5P8	Laccase	0 ± 0^a^	0.33 ± 0.06^b^	3.31 ± 0.19^c^	2.79 ± 0.29^c^
I6QS85	Laccase	0 ± 0^a^	0.42 ± 0.08^b^	2.88 ± 0.10^c^	2.68 ± 0.25^c^
Q50JG3	Laccase4	0 ± 0^a^	0.53 ± 0.06^b^	3.54 ± 0.17^c^	2.75 ± 0.15^c^
Q99056	Laccase‐5	0 ± 0^a^	0.46 ± 0.02^b^	2.78 ± 0.07^c^	2.47 ± 0.09^c^
A7U4S7	Peroxidase (Fragment)	0.68 ± 0.03^a^	0.52 ± 0.03^a^	3.96 ± 0.13^d^	2.68 ± 0.25^c^
O74640	Peroxidase	0.24 ± 0.02^a^	0.54 ± 0.03^a^	1.56 ± 0.02^b^	1.69 ± 0.07^b^
B9VR21	Mn Peroxidase	0.66 ± 0.11^a^	0.35 ± 0.02^a^	5.65 ± 0.15^b^	5.71 ± 0.05^b^
O74179	Mn Peroxidase	0.47 ± 0.04^a^	0.87 ± 0.04^a^	4.99 ± 0.11^b^	5.68 ± 0.22^c^
G8FPZ2	Mn Peroxidase	0.67 ± 0.09^a^	0.47 ± 0.02^a^	5.65 ± 0.17^c^	4.15 ± 0.03^b^
A0A060SDE3	Mn Peroxidase	0.42 ± 0.07^a^	0.55 ± 0.03^a^	5.97 ± 0.18^b^	5.24 ± 0.23^b^
A0A060SDU0	Mn Peroxidase	0.74 ± 0.03^a^	0.85 ± 0.04^a^	5.31 ± 0.19^b^	5.79 ± 0.29^b^
B5MAF4	Phenol oxidase	0.54 ± 0.02^a^	0.63 ± 0.03^a^	3.54 ± 0.17^b^	2.75 ± 0.15^b^
R7SQ98	Diphenol oxidase‐A2	0.24 ± 0.09^a^	0.88 ± 0.04^b^	2.78 ± 0.07^c^	2.47 ± 0.09^c^

Different superscript letters within column is significantly (*P* < 0.05) different.

Lignin has a highly branched three‐dimensional poly‐phenolic structure and can ultimately be converted into CO_2_ by microorganisms to maintain the carbon balance in nature (Houtman *et al*., 2018). Oxidoreductases play an important part in the biological degradation of lignin by microorganisms (Janusz *et al*., 2017; Martinez *et al*., 2017).Classical fungal oxidoreductases comprise ligninolytic peroxidases and multicopper oxidases, mainly laccases with different redox potentials, that can act on lignin‐derived products (Kersten and Cullen, 2014). The ligninolytic system of white‐rot fungi includes extracellular lignin peroxidase, MnP, alkyl‐aryl etherase and laccase. In addition, heme peroxidases, glucose oxidase, isoamyl alcohol oxidase, glutathione reductase, glutathione S‐transferase, copper‐radical oxidase, cellobiosedehydrogenase (CDH) and glucose/methanol/choline oxidase/dehydrogenase were also found to provide the hydrogen peroxide required by ligninolytic peroxidases. Here, Table 1 showed that a total of 25 lignin‐degrading enzymes providing auxiliary oxidoreductases were identified in the secretomes of *P. chrysosporium, I. lacteus, P. eryngii* and *P. ostreatus*. Among of these proteins, 8 laccases, 5 manganese peroxidases, 2 peroxidase, 1 diphenol oxidase, 3 glyoxal oxidases, 4 glucose–methanol–choline(GMC) oxidoreductases, 1 glyoxylate dehydrogenase and 1 phenol oxidase were identified as differentially expressed proteins. As expected, laccases were absent in the secretome of *P. chrysosporium*. MnP was the most highly expressed auxiliary oxidoreductases in the secretomes of *P. eryngii* and *P. ostreatus*, while it was less highly expressed in the secretomes of *P. chrysosporium* and *I. lacteus*. As shown in Table 1, compared with *P. chrysosporium* and *I. lacteus*, glyoxal oxidase, GMC oxidoreductase, laccase, MnP, glyoxylate dehydrogenase and phenol oxidase were more highly expressed in the *P. eryngii* and *P. ostreatus* secretomes cultivated on ramie stalk medium.

Although these ligninases were identified, at present, the complete degradation of natural lignin by a single enzyme cannot be realized. Enzymes related to the degradation of lignin can be divided into two types: (i) enzymes directly acting on lignin: including laccase, other related polyphenol oxidase, peroxidase and cellobiose dehydrogenase. (ii) Auxiliary enzymes collaboratively degrade lignin with enzymes directly acting on lignin: including aryl alcohol oxidase, vanillin alcohol oxidase and glyoxal oxidase (Anthony *et al*., 2013). Previous research has shown that a single laccase or manganese peroxidase does not have ideal activity for lignin degradation and that two enzymes can improve the lignin degradation ratio and that their synergistic coefficient can reach 1.464 (Janusz *et al*., 2013). Cellobiose dehydrogenase contains a flavin and a heme group. It can oxidize lignin by generating hydroxyl groups in Fenton‐type reaction in the presence of H_2_O_2_ and chelated iron ions. It was proposed to act on the reduction of quinones, which can be used by ligninolytic enzymes or in support of a MnP reaction. Glyoxal oxidases are copper‐radical oxidases with a broad specificity able to oxidize simple aldehydes to the corresponding carboxylic acids (Whittaker *et al*., 1996; Zamocky *et al*., 2006). However, the synergistic potentials of other sets of lignin‐degrading enzymes are still unclear. As we described above, seven ligninases were expressed at the same time in these secretomes, and although there might be synergistic interactions between these proteins, more experiments are needed to examine this question.

### Differentially expressed sugar transporters, cytochrome P450 monooxygenases and other proteins in the *P. chrysosporium, I. lacteus, P. eryngii* and *P. ostreatus* secretomes

In addition to secreting extracellular enzymes to degrade lignocellulose, the ability to metabolize wood polysaccharides depends on the transport and intracellular conversion of sugars. ABC transporters, major facilitator superfamily (MFS) transporters, phosphonate ABC transporter substrate‐binding proteins and metal ABC transporter permeases were also identified in the *P. chrysosporium, I. lacteus,P. eryngii and P. ostreatus* secretomes in this study (Table 2). But more interestingly, the ABC transporter and the MFS ransporter were more highly expressed in the *P. chrysosporium* and *I. lacteus* secretomes. This group comprises cellobiose transporters, supporting that the transporter proteins identified in the *P. chrysosporium* and *I. lacteus* secretomes could have a role in cellobiose and/or cellodextrin uptake.

**Table 2:** Differentially expressed sugar transporters in *P. chrysosporium*, *I. lacteus*, *P. eryngii* and *P. ostreatus* secretome.

Accession	Description	*P. chrysosporium*	*I. lacteus*	*P. eryngii*	*P. ostreatus*
R7S3H1	ABC protein	3.55 ± 0.18^b^	3.87 ± 0.02^b^	1.22 ± 0.06^a^	1.25 ± 0.06^a^
A0A0L6JDG5	ABC transporter	3.96 ± 0.20^b^	3.25 ± 0.16^b^	0.65 ± 0.04^a^	0.86 ± 0.04^a^
A0A0L6JAE4	ABC transporter permease	3.68 ± 0.15^b^	3.54 ± 0.17^b^	0.87 ± 0.05^a^	0.97 ± 0.06^a^
A0A0L6J367	ABC transporter substrate‐binding protein	3.67 ± 0.16^b^	3.55 ± 0.18^b^	0.58 ± 0.03^a^	0.74 ± 0.05^a^
A0A0L6JFC7	Metal ABC transporter permease	2.85 ± 0.13^b^	2.57 ± 0.13^b^	0.45 ± 0.02^a^	0.56 ± 0.03^a^
J0CZ65	MFS general substrate transporter	2.47 ± 0.12^b^	2.65 ± 0.13^b^	0.35 ± 0.02^a^	0.58 ± 0.03^a^
R7S6L7	MFS general substrate transporter	2.98 ± 0.18^b^	2.68 ± 0.10^b^	0.42 ± 0.03^a^	0.37 ± 0.01^a^
A0A0L6JEM5	MFS transporter	4.35 ± 0.22^c^	3.18 ± 0.16^b^	0.68 ± 0.03^a^	0.57 ± 0.03^a^

Different superscript letters within column is significantly (*P* < 0.05) different.

White‐rot fungi encode a large repertoire of P450 genes, which are known to be involved in the oxidation of phenolic and non‐phenolic aromatic compounds. The current proteomic analysis revealed that 7 cytochrome P450 monooxygenases were highly expressed in the *P. eryngii* and *P. ostreatus* secretomes. In addition, peptidases, proteinases and kinases were also quantified (Table 3). Carboxypeptidase and polyphosphate kinase were highly expressed in the *P. eryngii* and *P. ostreatus* secretomes. The secretion of proteases during lignocellulose degradation has previously been reported, which suggests that the proteases detected in cellulolytic cultures are correlated to the activation of cellulase activity and the cleavage of CDH functional domains (Baldrian and Valaskova, 2008). But the roles of peptidases, proteases and kinases in lignocellulose degradation are still not completely clear.

**Table 3:** Cytochrome P450 monooxygenases and other proteins.

Accession	Description	*P. chrysosporium*	*I. lacteus*	*P. eryngii*	*P. ostreatus*
R7SVV0	Cytochrome P450 (Fragment)	0.75 ± 0.01^a^	0.85 ± 0.03^a^	3.55 ± 0.18^b^	3.25 ± 0.17^b^
R7SIZ5	Cytochrome P450	0.54 ± 0.01^a^	0.52 ± 0.02^a^	3.65 ± 0.19^b^	3.98 ± 0.21^b^
R7SM57	Cytochrome P450	0.24 ± 0.01^a^	0.65 ± 0.03^a^	3.58 ± 0.18^b^	3.88 ± 0.20^b^
G5EJP0	Cytochrome P450	0.56 ± 0.02^a^	0.87 ± 0.04^a^	2.88 ± 0.12^b^	2.98 ± 0.17^b^
G5EJU7	Cytochrome P450	0.85 ± 0.04^a^	0.47 ± 0.01^a^	2.65 ± 0.10^b^	2.87 ± 0.13^b^
G5EJV5	Cytochrome P450	0.98 ± 0.06^a^	0.54 ± 0.03^a^	2.77 ± 0.10^b^	2.98 ± 0.15^b^
R7SI77	Cytochrome‐450 hydroxylase	0.55 ± 0.02^a^	0.68 ± 0.03^a^	2.68 ± 0.12^b^	2.49 ± 0.09^b^
R7SPH9	Family S53 protease	0.68 ± 0.04^a^	0.47 ± 0.01^a^	3.54 ± 0.18^b^	3.65 ± 0.16^b^
A0A0L6J1E5	carboxypeptidase	0.92 ± 0.05^a^	0.52 ± 0.02^a^	3.87 ± 0.20^c^	2.89 ± 0.15^b^
J0LJU0	P kinase	0.29 ± 0.15^a^	0.45 ± 0.17^a^	3.12 ± 0.13^b^	2.24 ± 0.29^b^

Different superscript letters within column are significantly (*P* < 0.05) different.

The current work describes for the first time the composition of the secretome of *P. chrysosporium, I. lacteus, P. eryngii* and *P. ostreatus* growing on ramie stalks. It was suggested that the knowledge on the different expressions of CAZyme was sufficient to discern the mechanisms implicated in selective ligninolysis of ramie stalks. Consistent with secretome analysis, cellulase activity was higher in *P. chrysosporium* and *I. lacteus*, while lignin‐degrading enzymes were higher in *P. eryngii* and *P. ostreatus*. The secretome of *P. eryngii and P. ostreatus* can be used for the pretreatment of straw lignocellulosic materials or to improve the efficiency of enzymatic saccharification through optimized enzyme–cocktails.

## Experimental procedures

### Microorganisms

*Phanerochaete chrysosporium* (CICC40934) and *P. eryngii* (CICC50126) were obtained from the China Center of Industrial Culture Collection. *I. lacteus* (CGMCC5.0809) was obtained from the China General Microbiological Culture Collection Center. *P. ostreatus* (bio‐67015) was obtained from the inquiry network for microbial strains of China. These four species were precultured on potato dextrose agar plates at 28°C for 7 days and were then used for ligninolysis of ramie stalks.

### Lignocellulosic degradation of ramie stalks

Air‐dried ramie stalks from the Institute of Bast Fiber Crops at the Chinese Academy of Agricultural Sciences were cut into small chips (400–800 μm mesh). Three grams of ramie stalks was added into 7 ml H_2_O, 0.1% Tween‐80, 4 mmol l^−1^ veratryl alcohol, 0.2 mmol l^−1^ Mn^2+^in 300‐ml Erlenmeyer flasks and sterilized at 121°C for 20 min. *P. chrysosporium* was cultured under 37°C, and pH value of substrate was adjusted to 4.5. *I. lacteus, P. eryngii* and *P. ostreatus* were cultured under 28°C, and pH value of substrates was adjusted to 5.5. Four pieces (approximately 40 mm^2^ each) of each precultured white‐rot fungus were inoculated separately into the substrates. These cultures were statically incubated for 7, 14, 21 or 28 days. All of the experiments were carried out in triplicate. The percentages of cellulose, hemicellulose, and lignin in the treated and untreated ramie stalks were determined according to methods of the Laboratory Analytical Procedure of the National Renewable Energy Laboratory (Sluiter *et al*., 2008). The lignin‐selective degradation coefficient was calculated as follows:

$$\text{Lignin}\;‐\;\text{selective degradation coefficient}=\frac{\text{Lignin degradation ratio}}{\text{Cellulose degradation ratio}}$$

### Enzymatic hydrolysis

Samples of treated or untreated ramie stalks (0.16 g) were suspended in citric acid buffer (pH4.8) and hydrolysed with 10–50 filter paper units (FPU) g^−1^ cellulase for 48 h at 50°C. FPU g^−1^ is the activity of the cellulase added per gram of stalk. After 48 h, the hydrolysis mixture was separated by centrifugation at 10 000 rpm for 5 min. Reducing sugar was measured using the 3,5‐dinitrosalicylic acid (DNS) reagent. The ratio of reducing sugar (mg g^−1^) was calculated as follows:

$$\text{Reducing sugar ratio (mg}/\text{g)}=\frac{\text{Amount of reducing sugar after enzymatic hydrolysis}}{\text{Amount of dry hemp woody core}}$$

### Enzyme assay

Ramie stalks colonized by mycelia were collected at different stages of the solid‐state fermentation (7–28 days) for time–course studies of enzyme activities. Extracellular enzymes were extracted as follows: 3 g wet weight of substrate was added to 50 ml of 50 mM citric acid buffer (pH 4.8) and stirred at 125 rpm for 5 h on an ice bath. The mixture was separated by centrifugation at 10 000 rpm for 5 min, and the supernatant was used for determination of enzyme activity. Carboxymethyl cellulase (CMCase), laccase and manganese peroxidase (MnP) activities were assayed as described in previous works (Kuhar *et al*., 2016; Vijayaraghavan *et al*., 2016; Jiang *et al*., 2018).

CMCase assays were performed in 50 mM citrate/phosphate buffer, at 50°C for 30 min using 1% w/v carboxymethyl cellulose (CMC) as the substrate. After 30 min, the reaction was stopped by adding 3 ml of DNS reagent, the mixture was boiled for 15 min cooled on ice, and optical density at 550 nm was determined. One unit of CMCase activity was defined as μmol glucose equivalents liberated min^−1^.

Laccase activity was measured using 2,2'‐azino‐bis (3‐ethylbenzothiazoline‐6‐sulfonic acid) (ABTS) as substrate. The rate of ABTS oxidation was determined at 420 nm. The reaction mixture contained 600 μl 0.1 M sodium acetate buffer (pH 5.0), 300 μl ABTS (5 mM), 300 μl culture filtrate and 1400 μl distilled water. The mixture was then incubated for 2 min at 30°C, and the absorbance was measured immediately at 1‐min intervals. One unit of laccase activity was defined as the conversion of 1 μmol of ABTS per minute.

MnP activity was measured as 2,6‐dimethoxyphenol (2,6‐DMP;ε = 27500 M^−1^ cm^−1^) oxidation. The reaction mixture contained 20 mM 2,6‐DMP, 20 mM MnSO_4_·H_2_O, 4 mM H_2_O_2_ and enzyme solution. The reaction was started by adding H_2_O_2_ and monitored by the absorbance at 469 nm. One unit (U) of enzyme activity was defined as the amount of MnP that oxidizes 1 μmol of 2,6‐DMP per minute at 30°C. The activities of laccase and MnP were expressed in units per litre. Enzyme activity results were the average of 20 replicates.

### Protein extraction, digestion, and iTRAQ labelling

Secretome analysis was performed using 10 culture bottles for each fungal species pooled together and labelled as sample A to minimize biological variation. Sample B was prepared in the same way as sample A, and each sample (A and B) was processed separately. Extracellular proteins were extracted from 10 g wet weight substrate incubated at 28°C for 21 days with 200 ml of 50 mM sodium acetate buffer (pH 5.0) and were stirred at 180 rpm for 1 h on an ice bath. The supernatants were collected by filtration through cheese cloth, centrifuged at 8000 *g* for 30 min at 4°C and passed through a 0.22‐μm cellulose acetate filter. The supernatants were then mixed with an equal volume of cold 40% trichloroacetic acid (TCA) and incubated on ice overnight. The precipitate was collected, washed with 20% TCA and washed with acetone twice. Proteins were then lyophilized.

Eight secretome samples from four white‐rot fungal species were analysed using iTRAQ technology according to the manufacturer's instructions (AB Sciex Inc., Framingham, MA). Protein samples (100 μg) were dissolved on ice in 100 μl buffer A containing 50 μl 8 M urea, 0.1 mg sodium dodecyl sulfate, 9 μl 500 mM triethyl ammonium bicarbonate and 40 μl ultrapure water. Then, 5 μl of 200 mM tris (2‐carboxyethyl) phosphine was added and the mixture was oscillated at 700 rpm at 55°C for 1 h. Reduction, alkylation and trypsin digestion of proteins were performed as described in the literature (Qin *et al*., 2013; Wang *et al*., 2013). Tryptic peptides were labelled using the iTRAQ 8Plex Multiplex Kit (Applied Biosystems, Foster City, CA, USA) according to the manufacturer’s protocol. Peptides from different fungal species were labelled as follows: 113 and 117 for *P. chrysosporium*, 114 and 118 for *I. lacteus*, 115 and 119 for *P. ostreatus*, and 116 and 121 for *P.eryngii*. After labelling, the eight labelled groups of peptides were mixed, lyophilized and re‐suspended in 2% H_3_PO_4_. The peptides were then purified by strong cation exchange chromatography and lyophilized as described in the literature (Guest, 2018).

### Mass spectrometric data search and analysis

Protein identification and quantification were performed as previously described in the literature (Qin *et al*., 2013; Wang *et al*., 2013). All MS analyses were performed on a LTQ Orbitrap Velos (Thermo Fisher Scientific, Bremen, Germany) connecting to an EASY‐nLC system via a nanospray source. The iTRAQ‐labelled peptides were dissolved in mobile phase A (99.9% H_2_O and 0.1% formic acid), and the samples (about 1 μg) were fractionated on a high‐performance liquid chromatography (HPLC) pre‐column(2 cm, ID 100 μm, 5 μm, C18) followed by an XBridge BEH130 Nano Ease column (15 cm, ID 75 μm, 3.5 μm, C18) with a flow rate of 300 nl min^−1^. The HPLC separation was performed with mobile phase A (0.1% acetic acid) and mobile phase B (98% acetonitrile, 0.5% acetic acid). The mobile phase B gradient was set as follows: 5% to 17% for 5 min, 17% to 25% for 90 min, 25% to 60% for 10 min, 60% to 80% for 5 min, and 80% buffer B for 10 min. The MS was operated in data‐dependent mode. MS/MS for each duty cycle was determined from a survey scan, and one full scan was analysed using the 25 most intense precursor ions for collision‐induced dissociation fragmentation (collision energy 35%). The intense precursor ions were detected in the Orbitrap analyser and selected for higher energy collision‐induced dissociation (HCD)‐MS^3^ fragmentation.

### Protein identification

Protein identification was performed using Maxquant (version 1.2.2.5) as described in the literature (Qin *et al*., 2013; Wang *et al*., 2013). The LC‐MS/MS data were queried against Uniprot. Our defined parameters were set as follows: (i) fixed modification, cysteine carbamidomethylation and iTRAQ modifications (N‐terminus and lysine residues); (ii) variable modification, methionine oxidation; (iii) precursor mass tolerance, ±20 ppm; 0.5‐dalton product ion mass tolerance; and (iv) trypsin digestion, up to two missed cleavages. Based on a target–decoy approach, low‐confidence peptides with a global false‐discovery rate (FDR) ≥ 1% were removed from further protein analysis. Relative protein abundance ratios between pairs of white‐rot fungal species were calculated from iTRAQ reporter ion intensities derived from HCD spectra. The median ratio in Maxquant was used to weight and normalize the quantitative ratios. Analysis of variance (ANOVA) was used to detect significant differences among groups, and a permutation‐based FDR value < 0.05 was considered significant.

### Statistical analysis

All of the experimental values presented in figures and tables are the mean ± standard deviation calculated using Excel 2007. Multiple comparison tests were performed using the *t*‐test with the Bonferroni correction (significance levels = 0.05).

## Conflict of interest

The authors declare no conflict of interest.

## Supporting information


**Table S1.** All of the identified proteins from the four white‐rot fungi cultivated on ramie stalks at 21 days.
**Table S2.** All of the identified proteins from *P. chrysosporium* cultivated on ramie stalks at 21 days.
**Table S3.** All of the identified proteins from *Irpex lacteus* cultivated on ramie stalks at 21 days.
**Table S4.** All of the identified proteins from *P. eryngii* cultivated on ramie stalks at 21 days.
**Table S5.** All of the identified proteins from *P. ostreatus* cultivated on ramie stalks at 21 days.
